# Patient perspectives on naloxone receipt in the emergency department: a qualitative exploration

**DOI:** 10.1186/s12954-022-00677-7

**Published:** 2022-08-26

**Authors:** Margaret Lowenstein, Hareena K. Sangha, Anthony Spadaro, Jeanmarie Perrone, M. Kit Delgado, Anish K. Agarwal

**Affiliations:** 1 Division of General Internal Medicine, Perelman School of Medicine, University of Pennsylvania, 122 Blockley Hall, 421 Guardian Drive, Philadelphia, PA 19104 USA; 2 Leonard Davis Institute of Health Economics, University of Pennsylvania, Philadelphia, PA USA; 3 Center for Addiction Medicine and Policy, University of Pennsylvania, Philadelphia, PA USA; 4 Department of Emergency Medicine, Perelman School of Medicine at the University of Pennsylvania, Philadelphia, PA USA

**Keywords:** Naloxone, Overdose prevention, Harm reduction, Emergency departments

## Abstract

**Background:**

Emergency departments (EDs) are important venues for the distribution of naloxone to patients at high risk of opioid overdose, but less is known about patient perceptions on naloxone or best practices for patient education and communication. Our aim was to conduct an in-depth exploration of knowledge and attitudes toward ED naloxone distribution among patients who received a naloxone prescription.

**Methods:**

We conducted semi-structured telephone interviews with 25 adult participants seen and discharged from three urban, academic EDs in Philadelphia, PA, with a naloxone prescription between November 2020 and February 2021. Interviews focused on awareness of naloxone as well as attitudes and experiences receiving naloxone in the ED. We used thematic content analysis to identify key themes reflecting patient attitudes and experiences.

**Results:**

Of the 25 participants, 72% had previously witnessed an overdose and 48% had personally experienced a non-fatal overdose. Nineteen participants (76%) self-disclosed a history of substance use or overdose, and one reported receiving an opioid prescription during their ED visit and no history of substance use. In interviews, we identified wide variability in participant levels of knowledge about overdose risk, the role of naloxone in reducing risk, and naloxone access. A subset of participants was highly engaged with community harm reduction resources and well versed in naloxone access and use. A second subset was familiar with naloxone, but largely obtained it through healthcare settings such as the ED, while a final group was largely unfamiliar with naloxone. While most participants expressed positive attitudes about receiving naloxone from the ED, the quality of discussions with ED providers was variable, with some participants not even aware they were receiving a naloxone prescription until discharge.

**Conclusions:**

Naloxone prescribing in the ED was acceptable and valued by most participants, but there are missed opportunities for communication and education. These findings underscore the critical role that EDs play in mitigating risks for patients who are not engaged with other healthcare or community health providers and can inform future work about the effective implementation of harm reduction strategies in ED settings.

**Supplementary Information:**

The online version contains supplementary material available at 10.1186/s12954-022-00677-7.

## Introduction

North America is in the midst of a persistent overdose crisis, with the majority of overdose deaths involving opioids [1, 2]. After briefly plateauing, there have been substantial increases in overdose death rates during the COVID-19 pandemic, with data demonstrating the highest overdose death rate ever [3]. A key strategy for reducing overdose death is the wider distribution of the overdose reversal agent naloxone [4]. Laypeople can safely and effectively administer naloxone to reverse overdose, and community education with naloxone distribution is associated with decreased overdose mortality [5–7]. The World Health Organization endorses provision  of take-home naloxone programs [8], in which naloxone is distributed to potential bystanders of an overdose in the community, and there is evidence from across the world that these programs can be safely and effectively implemented [9–11]. Globally, most take-home naloxone distribution occurs via syringe service programs and other community-based programs serving people who use drugs [12, 13]. However, distribution of naloxone to at-risk patients through medical settings is another strategy for more widespread naloxone distribution that may reach a broader population. In the USA, both medical and non-medical naloxone distribution models have been adopted, but distribution in medical settings—typically via a prescription—is still limited [6, 14].

Emergency departments (EDs) are important venues to identify and engage high-risk individuals in overdose prevention. Patients present to EDs for a multitude of reasons associated with opioid use disorder (OUD) including following an overdose and for complications of their substance use; thus, EDs provide an opportunity to engage with patients who may not otherwise access regular care [15, 16]. Recent US guidelines have called for naloxone distribution to become the standard of care in ED OUD management [16, 17]. Despite the promise, real-world implementation challenges remain in getting naloxone to patients, especially those at the highest risk. The majority of patients at-risk for overdose do not receive naloxone prescriptions [14], including patients with OUD-related ED visits [18], with less than 1% of US patients filling naloxone prescriptions.

Investigating patient attitudes and perspectives is important in motivating behavior change and informing pragmatic action. While there are several studies examining patient attitudes toward overdose education and naloxone distribution in primary care settings, particularly in patients on chronic opioid therapy [19–21], less is known about attitudes in ED patients. Much of the work related to naloxone in the ED setting has focused on tools to better identify patients and facilitate appropriate prescribing [22, 23] and direct ED naloxone provision of take-home naloxone [24, 25]. A qualitative study of overdose survivors in the ED highlighted the potential of EDs as a source of important resources, particularly overdose prevention education and naloxone [26]. Participants emphasized the importance of provider communication as critical to their experience. However, gaps persist in the understanding of how to move toward best practices for discussing naloxone prescribing and distribution within the ED.

The aim of this study was to conduct an in-depth exploration of patient knowledge and attitudes toward ED naloxone distribution in a sample of patient participants who received an ED naloxone prescription. These results can inform future studies around best practices and approaches for ED-based naloxone prescribing and education.

## Methods

Our study included semi-structured qualitative interviews of patient participants who received take-home naloxone (either prescribed or dispensed in the ED) following their ED visit. Interviews explored patient experiences with naloxone prescribing in the ED, including knowledge and attitudes, communication with providers, and ability to obtain the prescription. This study followed the Consolidated Criteria for Reporting Qualitative Research (COREQ) [27].

### Study setting and time period

Participants were recruited from two urban EDs within a large, academic health system in Philadelphia. Philadelphia is highly impacted by OUD and overdose, with the highest overdose death rate among the largest US cities [28]. Study EDs included a large tertiary referral hospital and a level 1 trauma center, which together see over two thousand OUD-related visits annually. The study EDs had previously implemented a robust program of quality improvement for ED-based OUD care, including the availability of guidelines and order sets that incorporate OUD treatment and naloxone prescribing for patients at risk of overdose [29]. Study EDs are also supported by peer recovery specialists well-versed in harm reduction. Interviews were conducted between November 2020 and February 2021.

### Selection of participants

Criteria for inclusion in the study were: any adults (18 years of age or older) who were treated and discharged from study EDs who received a prescription for naloxone upon discharge during the study period. Naloxone prescribing was based on provider discretion, and we did not have access to information about the rationale for a naloxone prescription. In deciding to prescribe naloxone, ED providers had access to guidelines and order sets that recommended naloxone prescription for patients at risk for an overdose, including those with OUD or people who use substances that might contain fentanyl or other opioids. Providers placed naloxone orders in the electronic medical record. One of the EDs dispenses take-home naloxone to patients prescribed naloxone at discharge, while the other ED exclusively prescribes naloxone to be picked up at a pharmacy, and we included patients from both. Participants were excluded if they were pregnant or incarcerated or if they did not have access to a phone as interviews were conducted by phone.

Potential participants were identified by electronic health record review based on receipt of a discharge prescription for naloxone. Participants were not explicitly asked about the reason for naloxone receipt, whether due to OUD, overdose, opioid prescription, or other indication, although they were free to disclose this in their interview responses. All eligible participants were contacted using secure, HIPAA-compliant text messaging through the WayToHealth platform [30]. Participants answered a brief series of prompts confirming their receipt of naloxone and inquiring about their interest in participating in a longer interview. A consecutive sample of those who expressed interest were contacted by telephone to schedule a telephone interview. Participants were compensated $50 for completing the interviews.

Prior to enrollment, the research staff verified inclusion and exclusion criteria. All participants completed an informed consent process with study staff that detailed the study purpose, protocol, and potential risks and benefits.

### Interview guide

The interview guide focused on participant attitudes toward naloxone generally and in the ED (see Additional file 1). Literature on patient perceptions of naloxone for OUD is limited, so interview prompts drew from prior literature on patients’ attitudes toward naloxone in other populations or settings [20, 31, 32]. Topics included (1) general awareness of naloxone, (2) experiences obtaining naloxone, and (3) attitudes about receiving naloxone. The interview guide was developed and piloted with input from members of the research team, including emergency medicine and addiction medicine physicians and research assistants, as well as a person with lived experience with OUD and another with experience in harm reduction advocacy. We also collected demographic information and asked whether participants had obtained the naloxone they were prescribed and whether they carried and/or used the naloxone.

The interviews were conducted by a trained BA-level research assistant (HKS). Researchers had no preexisting relationships with interviewees. Interviews ranged from 10 to 30 min, and a total of 25 interviews were conducted, at which point the research team determined that thematic saturation was reached.

### Data analysis

Interviews were recorded and transcribed verbatim using a professional transcription service. We developed a coding structure based on concepts from the interview guide as well as emergent themes. Two trained research assistants used thematic content analysis to code all of the transcripts using NVivo 11.0 software (QSR International) [33]. 20% of transcripts were double-coded, with high interrater reliability (mean *κ* = 0.93). Then, the research team collaborated in group discussions, iterative interpretation of the data, and the final analysis.

This study was approved by the University of Pennsylvania Institutional Review Board.

## Results

### Participant characteristics

Two hundred and five participants were contacted by text following their ED visit, and 40 responded to the texting intervention. Of the 40 participants reached, 25 consented to the interview. The mean age was 37 years and participants were majority male, stably housed and publicly insured (Table 1). 48% self-identified as Black or African American, and 40% were White. Almost half (48%) reported a prior non-fatal overdose, and the majority (72%) had previously witnessed an overdose. Phone ownership was required for participation, and the majority (96%) had a smartphone. Nineteen participants self-disclosed a history of OUD or overdose, five participants offered no information about the diagnosis, and one participant reported receiving an opioid prescription during the ED visit along with naloxone and no history of substance use.

**Table 1:** Participant characteristics

Characteristic	Study group (*n* = 25)
Age, mean (SD)	37 (11)
*Gender, no. (%)*	
Male	15 (60)
*Highest level of education, no. (%)*	
High school/GED or less	9 (36)
Associate degree or some college	8 (32)
Bachelor’s degree	6 (24)
Unknown/not reported	2 (8)
*Race, no. (%)*	
Black or African American	12 (48)
White	10 (40)
More than one race	3 (8)
*Ethnicity, no. (%)*	
Hispanic or Latino	2 (8)
*Housing status, no. (%)*	
Permanent housing, stable	18 (72)
Recovery house	2 (8)
Unstably housed	4 (16)
Unknown/not reported	1 (4)
*Insurance status, no. (%)*	
Medicaid	18 (72)
Commercial	4 (16)
Medicare	2 (4)
Uninsured	1 (4)
*Phone status, no. (%)*	
Has access to a smartphone	24 (96)
Has access to a mobile phone (not a smartphone)	1 (4)
*Personal history of overdose, no. (%)*	
Has previously overdosed	12 (48)
*Witnessed an overdose*	
Has previously witnessed an overdose	18 (72)

### Interview themes

#### Variable knowledge and awareness about naloxone prior to ED encounters

Among participants, we found wide variation in patient understanding and knowledge about naloxone (Table 2). Participants with explicit OUD-related presentations (overdose or other visits) were found among all groups, including those with limited familiarity with naloxone and harm reduction interventions. The first subset of participants was savvy about accessing and using naloxone and able to describe multiple ways of obtaining it, including healthcare settings, pharmacies, and community groups. These participants also expressed familiarity with local harm reduction organizations where they could obtain naloxone along with other resources and high levels of confidence in their ability to respond to an overdose. Finally, participants in this group reported carrying naloxone regularly, seeing the ED encounter as an opportunity to “stock up” for themselves and others. For this group, the ED was one of many potential sources of the medication, and receiving a prescription for naloxone in the ED was a familiar and expected part of their care.

**Table 2:** Key themes and supporting quotations

Variable knowledge and awareness about naloxone	
*Subset of participants with high levels of knowledge about naloxone access and high engagement with community harm reduction resources*	
Awareness about multiple sources for naloxone access	"You could go to the emergency room, a pharmacy or needle exchange." (Participant 12)
Engagement with community harm reduction resources	"They had the program started up where they were giving me… the Narcan and they was giving me different things, the free needles, the alcohol swabs and stuff like that. I would disperse it through the neighborhood. I was around some people that were greedy so, whenever they would go to get high, I would follow them to see if they were going to OD and I would Narcan them. I only Narcaned one person and I had someone Narcan me once." (Participant 5)
High levels of confidence about naloxone access and use	"[Community organization] always make sure you’re telling us something about something. So we never go there and don’t know anything. They’re always helping us, keeping us on point. I think it’s just a little overkill at this point. People should know already, why you need [naloxone] and the severity of it." (Participant 1)
*Subset of participants with familiarity with naloxone but limited engagement with community harm reduction organizations and resources*	
Healthcare encounters were primary source of naloxone	“I’m on Suboxone in a small program and every time I get a small [prescription] of Suboxone, they give me a box of Narcan.” (Participant 16)
Familiar with naloxone through friends or social networks	“I was given Narcan before, just through friends, people I knew who gave it to me, but that was the first time me getting prescribed it through the emergency room” (Participant 6)
Dealers as a source for naloxone	“Ordinarily, if I go buy first it’s the heroin. I’ll then ask who has a little shot of [naloxone]. Sometimes they have it or they will give me the resources to go get it. Some people actually are selling it out there” (Participant 9)
*Subset of participants with limited knowledge about overdose risk, overdose prevention, and naloxone access prior to ED encounter*	
Low awareness of overdose risk	“I never went looking for [naloxone] because I never thought I needed it” (Participant 17)
Limited knowledge about insurance coverage	“I never got [naloxone] because I didn’t know if my insurance paid for it or not” (Participant 13)
Lack of knowledge about naloxone and its role in reducing overdose risk	“People don’t know that [naloxone] exists until a doctor tells you about it. There’s nothing to find, or nothing around promoting it or anything. It’s just like a secret between the ER and different persons from the ER” (Participant 19)

Attitudes toward receipt of naloxone in the ED	
Positive sentiments toward receiving naloxone in the ED	“I feel they were looking out for my best interest. They gave it to me just in case.” (Participant 2)
Naloxone as overdose prevention strategy	I don’t think that I’m somebody who is at risk for overdose … but ultimately, I understand the purpose of prescribing it to somebody, anybody who’s been under so much, who had so much medication” (Participant 4)
Sense of empowerment	“I thought [the naloxone prescription] was helpful. Now I can change someone’s life if I needed to.” (Participant 13)
Potential to feel stigmatized	“I felt insulted because I was like ‘Damn, what do I need Narcan for?’” (Participant 23)

Communication with ED clinicians about naloxone	
Lack of communication about naloxone prescription	“I did not know that they were going to prescribe [naloxone] to me. They never said anything about it. I just found out, I think, when I looked at my discharge papers” (Participant 4)
	“I was in the ED because of some other issues that I have with chronic pain and I found out. Well, I didn’t actually know that they were giving me Narcan or a prescription for Narcan until I got my list of prescriptions at discharge. So, I didn’t really know about it” (Participant 24)
Lack of communication about overdose risk	“I told them I was going through withdrawal and they, but nonetheless, they gave me a box of [naloxone]” (Participant 16)
	“Well, say if you go in there for a different reason for what I went, I understand about it, because I’ve been on fentanyl. Other people might not be able to understand it and might take it as a harsh gesture” (Participant 19)

The second subset of participants expressed some familiarity with naloxone but lower awareness about naloxone access and less engagement with community harm reduction resources. This group reported obtaining naloxone primarily through various touchpoints within the healthcare system, whether through primary care, outpatient substance use programs, or their ED visit. Some reported familiarity with naloxone through friends, family, or acquaintances, but overall there was less knowledge about how to acquire naloxone outside of healthcare encounters, such as through community organizations or at pharmacies without a prescription through a standing order in the state of Pennsylvania [34]. A few participants reported purchasing naloxone on the street from dealers as a precaution along with the substances they used regularly. This second subset of participants who desired naloxone but were less knowledgeable or engaged with community supports viewed the ED as an important resource for naloxone access and one of the few opportunities for overdose prevention interventions.

The final subset of participants reported little to no knowledge about naloxone prior to their ED encounters. Although many reported presenting for OUD or overdose-related reasons, among this group there was a lack of awareness of what naloxone was, their personal overdose risk, and strategies to mitigate this risk. There was also limited knowledge about how to obtain and use naloxone or that the medication was covered by insurance. Despite the prevalence of naloxone and overdose prevention interventions through the city described by some participants, for this group the ED visit was sometimes their first time receiving naloxone or other overdose prevention counseling.

#### Attitudes toward receipt of naloxone in the ED

Among participants, the majority expressed positive sentiments toward receipt of naloxone in the ED. Many identified naloxone prescribing as an important safety precaution for reducing overdose deaths and believed that naloxone should be free and widely accessible. Not all participants perceived that they were at risk for overdose, but there was a recognition of the importance of naloxone access to reduce overdoses in their community. Possession of naloxone created a sense of empowerment among participants who carried it regularly and valued it as a tool to help their community. A minority of participants highlighted the potential for negative feelings about receiving a naloxone prescription in the ED, largely due to the stigma associated with drug use and addiction. A few cautioned that a naloxone prescription might be taken the wrong way by other patients, particularly those that came to the ED for reasons that were not substance use related.

#### Communication with ED clinicians about naloxone

Given the variable levels of knowledge and experiences with naloxone prescribing, we explored patient experiences and perceptions of communication with ED clinicians specific to naloxone. One critical gap was a lack of communication about the naloxone prescription itself, with multiple participants reporting that their doctor or nurse had not told them that they would be receiving naloxone to take home or via prescription. Among participants who presented with painful conditions or who did not identify as having OUD, there was some confusion about naloxone co-prescribing when it was not discussed by a provider. Among participants who knew or were aware of their naloxone prescription, there were still misunderstandings about their personal overdose risk and the reason for the prescription. Finally, participants reported little about discussions of overdose risk, safer use, or carrying or using naloxone.

## Discussion

This study deployed qualitative methods to explore patient perspectives about receiving a naloxone prescription or being dispensed naloxone upon discharge from the ED. The results highlight a wide range among participants in their knowledge of overdose risk, the role of naloxone in reducing risk, and naloxone access. Participants were largely appreciative of naloxone prescribing, but there were varying levels of discussion with clinicians regarding the prescription. These patient-centered themes demonstrate the critical role played by EDs in distribution and education about naloxone, particularly for the subset of participants with limited engagement with the community or other settings where overdose prevention occurs. The results also highlight real-world, and high-impact, missed opportunities for communication and education with individuals about their own overdose risks and strategies to mitigate risks.

EDs represent a promising venue for interventions targeting OUD because patients present there for overdoses or other complications of substance use. Randomized controlled trial findings demonstrate that ED-initiated buprenorphine more than doubles treatment retention at 30 days compared with referral alone [35], and there have been calls to expand this practice [36]. However, not all patients with OUD will desire treatment initiation and even those on treatment remain at risk for overdose, so it is critical for OUD interventions in EDs and elsewhere to incorporate harm reduction strategies including naloxone distribution and counseling on safer use. Take-home naloxone programs have been shown to be safe and are associated with reductions in overdose deaths, particularly with training on overdose prevention [9–11]. US guidelines for ED OUD management have highlighted the importance of naloxone prescribing for those with OUD [16]. Adoption of ED-based naloxone distribution is less common outside North America, although there have been promising pilot programs [37–39]. Our findings suggest that while efforts to boost prescribing of naloxone are important, these efforts will be most successful when paired with effective patient education and communication. The results presented here highlight several important findings that can inform practices of ED naloxone prescribing and distribution.

First, naloxone prescribing in the ED should be specifically coupled with a clear conversation about the prescription and basic education on overdose prevention. In our study, not all participants were even aware they would be receiving naloxone and others reported little to no discussion about overdose risk and safer use. Although overdose education should not be a prerequisite for naloxone receipt, an ED visit provides the ideal opportunity for a teachable moment which, for some, may be their only interaction with healthcare or harm reduction services. Naloxone distribution programs in community settings often couple teaching on safer substance use strategies, such as not using alone, using slowly or using a test dose, testing for fentanyl, and ensuring that others around them know how to use naloxone [6, 40]. Our results demonstrate substantial heterogeneity among participants in their levels of knowledge about naloxone and overdose risk and would benefit from education about these practices. Clinicians may feel less comfortable with harm reduction practices than they are with other aspects of medical care [41], and incorporating new practices in busy acute care settings can be challenging given the many competing demands [42]. Implementation of ED naloxone prescribing or distribution may need to be augmented with harm reduction teaching to medical staff, development of patient education materials, or other means of facilitating harm reduction conversations with patients. There are examples in the literature of algorithms designed to automate or “nudge” the provision of naloxone to at-risk ED patients [23]. While this may increase naloxone distribution, it does not guarantee a conversation or discussion. Other settings have incorporated staff with specific knowledge or lived experience to conduct this training, which may overcome some of the barriers related to time or knowledge but means that harm reduction continues to be separate from the rest of medical care [24]. Coupled with the literature, our results suggest that the implementation of high-quality interventions to promote uptake of treatment and harm reduction interventions in EDs will likely require multiple components.

Despite gaps in knowledge and communication, our results demonstrate that naloxone prescribing in the ED was generally acceptable to participants. While the literature on patient perceptions of naloxone prescriptions is limited, our findings mirror prior studies of overdose survivors in the ED and patients in the primary care setting [20, 26]. Participants in our study expressed a feeling of being “*looked out for*” by clinicians who provided naloxone as well as feeling empowered about their ability to save other people’s lives in their community. Strategies for ED communication about naloxone should harness these positive sentiments to focus on empowering individuals who use drugs to understand and reduce their own risk and the risks of those in their community and to clearly communicate that harm reduction is an important part of medical care. Patient education on risk reduction is even more critical in the era of illicit fentanyl contamination, with fentanyl present not only in heroin but also in stimulants and counterfeit prescription pills. [43]

Finally, substance use and overdose remain stigmatized, so it is critical that naloxone prescribing and distribution interventions in healthcare settings incorporate stigma mitigation strategies. Among the minority of participants in our study who expressed negative feelings about receiving naloxone in the ED, most related a sense that their needs were misunderstood or that they felt judged or ashamed by providers’ assumptions about their drug use. These participants’ perceptions reflect broader stigma toward substance use as well as current or past experiences of stigma in healthcare settings like the ED [26, 44]. Stigmatizing interactions in health care have also been cited as a barrier to receiving naloxone at pharmacies [32] and to care-seeking more broadly among people who use drugs [45, 46]. Despite these challenges, positive patient perceptions toward naloxone receipt in our study suggest that the provision of harm reduction care could be one way to foster trust in the healthcare system or providers and this deserves further study.

Our study has several important limitations. Our participants were recruited from two EDs in a single city so the results may not be transferable to all ED patients receiving naloxone. As is typical in qualitative methodologies, our study was not intended to generate quantitative information about the association of patient or clinical characteristics and the views expressed. In addition, we conducted interviews via telephone, which limited participation to individuals with access to a phone and who were willing to be interviewed in this venue. Many patients in this population have unstable housing or phone access, and this group may not be fully represented in these data. We also do not have information about patient diagnoses, the indication for the naloxone (OUD, overdose, opioid prescription) the clinical encounters, what was discussed with clinicians, or whether patients received naloxone in-hand versus a prescription, so our results only reflect the perceptions of participants about the provider communication and the clinical care that occurred. Although most participants disclosed OUD or overdose, some of the participants may have received naloxone for primary prevention in the case of opioid prescriptions. However, this would still merit discussion with the provider even if different from a conversation for a person with active OUD. Finally, one of the EDs serves as an opioid center for excellence in the state and has a robust research and clinical community dedicated to addiction and substance use treatment.

## Conclusions

In conclusion, we found that naloxone prescribing in the ED was acceptable and valued by most participants, but that there were missed opportunities for communication and education around naloxone prescribing and overdose prevention. These findings are important in informing future work about effective implementation of harm reduction strategies in ED settings and underscore the critical role that EDs play in mitigating risks for patients who are not engaged with other healthcare or community health providers.

## Supplementary Information

**Additional file 1**. Interview guide.

## Data Availability

The datasets generated and/or analyzed during the current study are not publicly available due to the nature of the study (interviews) but are available from the corresponding author on reasonable request.

## References

[1] Centers for Disease Control and Prevention. Understanding the epidemic. Centers for disease control and prevention, national center for injury prevention and control. https://www.cdc.gov/drugoverdose/index.html (2020). Accessed 7 Jul 2021.

[2] McNeil R, Jauffret-Roustide M, Hansen H (2022). Reducing drug-related harms and promoting health justice worldwide during and after COVID-19: an AJPH supplement. Am J Public Health.

[3] Centers for Disease Control and Prevention. Increase in fatal drug overdoses across the United States driven by synthetic opioids before and during the COVID-19 pandemic. https://emergency.cdc.gov/han/2020/han00438.asp (2020). Accessed October, 2021.

[4] Centers for Disease Control and Prevention. Overdose prevention. https://www.cdc.gov/drugoverdose/prevention/index.html (2017). Updated 31 Aug 2017. Accessed 15 Dec 2018.

[5] Walley AY, Xuan Z, Hackman HH (2013). Opioid overdose rates and implementation of overdose education and nasal naloxone distribution in Massachusetts: interrupted time series analysis. BMJ.

[6] Kerensky T, Walley AY (2017). Opioid overdose prevention and naloxone rescue kits: what we know and what we don’t know. Addict Sci Clin Pract.

[7] Fairbairn N, Coffin PO, Walley AY (2017). Naloxone for heroin, prescription opioid, and illicitly made fentanyl overdoses: challenges and innovations responding to a dynamic epidemic. Int J Drug Policy.

[8] World Health Organization (2014). Community management of opioid overdose.

[9] Clark AK, Wilder CM, Winstanley EL (2014). A systematic review of community opioid overdose prevention and naloxone distribution programs. J Addict Med.

[10] Giglio RE, Li G, DiMaggio CJ (2015). Effectiveness of bystander naloxone administration and overdose education programs: a meta-analysis. Inj Epidemiol.

[11] McDonald R, Strang J (2016). Are take-home naloxone programmes effective? Systematic review utilizing application of the Bradford Hill criteria. Addiction.

[12] Dwyer R, Olsen A, Fowlie C (2018). An overview of take-home naloxone programs in Australia. Drug Alcohol Rev.

[13] European Monitoring Centre for Drugs and Drug Addiction. Take-home naloxone. https://www.emcdda.europa.eu/publications/topic-overviews/take-home-naloxone#section6. Accessed 10 Aug 2022.

[14] Follman S, Arora VM, Lyttle C, Moore PQ, Pho MT (2019). Naloxone prescriptions among commercially insured individuals at high risk of opioid overdose. JAMA Netw Open.

[15] Adams JM (2018). Increasing naloxone awareness and use: the role of health care practitioners. JAMA.

[16] Hawk K, Hoppe J, Ketcham E (2021). Consensus recommendations on the treatment of opioid use disorder in the emergency department. Ann Emerg Med.

[17] Samuels EA, D'Onofrio G, Huntley K (2019). A quality framework for emergency department treatment of opioid use disorder. Ann Emerg Med.

[18] Kilaru AS, Liu M, Gupta R (2021). Naloxone prescriptions following emergency department encounters for opioid use disorder, overdose, or withdrawal. Am J Emerg Med.

[19] Behar E, Bagnulo R, Coffin PO (2018). Acceptability and feasibility of naloxone prescribing in primary care settings: a systematic review. Prev Med.

[20] Behar E, Rowe C, Santos GM, Murphy S, Coffin PO (2016). Primary care patient experience with naloxone prescription. Ann Fam Med.

[21] Mueller SR, Koester S, Glanz JM, Gardner EM, Binswanger IA (2017). Attitudes toward naloxone prescribing in clinical settings: a qualitative study of patients prescribed high dose opioids for chronic non-cancer pain. J Gen Intern Med.

[22] Funke M, Kaplan MC, Glover H (2021). Increasing naloxone prescribing in the emergency department through education and electronic medical record work-aids. Jt Comm J Qual Patient Saf.

[23] Marino R, Landau A, Lynch M, Callaway C, Suffoletto B (2019). Do electronic health record prompts increase take-home naloxone administration for emergency department patients after an opioid overdose?. Addiction.

[24] Samuels EA, Baird J, Yang ES, Mello MJ (2019). Adoption and utilization of an emergency department naloxone distribution and peer recovery coach consultation program. Acad Emerg Med Off J Soc Acad Emerg Med.

[25] Chen Y, Wang Y, Nielsen S, Kuhn L, Lam T (2020). A systematic review of opioid overdose interventions delivered within emergency departments. Drug Alcohol Depend.

[26] Hawk K, Grau LE, Fiellin DA (2021). A qualitative study of emergency department patients who survived an opioid overdose: perspectives on treatment and unmet needs. Acad Emerg Med Off J Soc Acad Emerg Med.

[27] Tong A, Sainsbury P, Craig J (2007). Consolidated criteria for reporting qualitative research (COREQ): a 32-item checklist for interviews and focus groups. Int J Qual Health Care.

[28] Philadelphia Department of Public Health. 2016 Overdoses From Opioids in Philadelphia. CHART Web site. http://www.phila.gov/health/pdfs/chart%20v2e7.pdf (2017). Accessed Oct 2021.

[29] Lowenstein M, Perrone J, Xiong RA (2021). Sustained implementation of a multicomponent strategy to increase emergency department-initiated interventions for opioid use disorder. Ann Emerg Med.

[30] Asch DA, Volpp KG (2012). On the way to health. LDI Issue Brief.

[31] Khatiwoda P, Proeschold-Bell RJ, Meade CS, Park LP, Proescholdbell S (2018). Facilitators and barriers to naloxone kit use among opioid-dependent patients enrolled in medication assisted therapy clinics in North Carolina. N C Med J.

[32] Donovan E, Case P, Bratberg JP (2019). Beliefs associated with pharmacy-based naloxone: a qualitative study of pharmacy-based naloxone purchasers and people at risk for opioid overdose. J Urban Health Bull N Y Acad Med.

[33] Pope CM (2000). Nicholas, qualitative research in health care.

[34] Levine R. Naloxone prescription for overdose prevention. In: Health Do, 2018.

[35] D’Onofrio G, O'Connor PG, Pantalon MV (2015). Emergency department-initiated buprenorphine/naloxone treatment for opioid dependence: a randomized clinical trial. JAMA.

[36] Martin A, Mitchell A, Wakeman S, White B, Raja A (2017). Emergency department treatment of opioid addiction: an opportunity to lead. Acad Emerg Med Off J Soc Acad Emerg Med.

[37] Black E, Monds LA, Chan B (2022). Overdose and take-home naloxone in emergency settings: a pilot study examining feasibility of delivering brief interventions addressing overdose prevention with ‘take-home naloxone’ in emergency departments. Emerg Med Australas.

[38] Jones M, Bell F, Benger J (2020). Protocol for take-home naloxone in multicentre emergency (TIME) settings: feasibility study. Pilot Feasibility Stud.

[39] Strang J, McDonald R, Campbell G (2019). Take-home naloxone for the emergency interim management of opioid overdose: the public health application of an emergency medicine. Drugs.

[40] Harm Reduction Coalition. Overdose Risks and Prevention. Opioid Overdose Basics Training Guide Web site. https://harmreduction.org/issues/overdose-prevention/overview/overdose-basics/opioid-od-risks-prevention/ (2020). Accessed 12 Jul 2021.

[41] Sue KL, Fiellin DA (2021). Bringing harm reduction into health policy—combating the overdose crisis. N Engl J Med.

[42] Lowenstein M, Kilaru A, Perrone J (2019). Barriers and facilitators for emergency department initiation of buprenorphine: a physician survey. Am J Emerg Med.

[43] Khatri UG, Viner K, Perrone J (2018). Lethal fentanyl and cocaine intoxication. N Engl J Med.

[44] van Boekel LC, Brouwers EP, van Weeghel J, Garretsen HF (2013). Stigma among health professionals towards patients with substance use disorders and its consequences for healthcare delivery: systematic review. Drug Alcohol Depend.

[45] Motavalli D, Taylor JL, Childs E (2021). “Health is on the back burner:” multilevel barriers and facilitators to primary care among people who inject drugs. J Gen Intern Med.

[46] Biancarelli DL, Biello KB, Childs E (2019). Strategies used by people who inject drugs to avoid stigma in healthcare settings. Drug Alcohol Depend.

