# Supplementary material for: Patient perspectives on naloxone receipt in the emergency department: a qualitative exploration
Source: Harm Reduct J. 2022 Aug 26;19:97. doi: 10.1186/s12954-022-00677-7 (PMC9412772; doi:10.1186/s12954-022-00677-7)
Supplement: Supplementary file 1 — Additional file 1. Interview guide. [file 12954_2022_677_MOESM1_ESM.docx]

**APPENDIX:**

1. **Do you currently carry naloxone?**

*Yes, no, sometimes*

*Tell me more about that … (good probe for all of these questions)*

1. **Tell me about your experiences getting naloxone.**

Probe:

- *ED visit, prior to ED visit, where did you get it*
- *Any challenges*
- *Any tips for making it easier*
- *How did you feel?*

1. **We provide Narcan to people who we think are at risk for overdose. We also encourage people at risk for overdose to discuss naloxone and its use with their family or other support network in the event that they needed to use Narcan for an overdose emergency. Tell me about your family/support system’s familiarity with Narcan.**

- *Do they know how to use it?*
- *Do they carry it?*
- *What are their attitudes about keeping Narcan/Naloxone nearby?*

1. **Are you currently carrying naloxone?**

*Yes, no*

*How often do you carry it? (days of the week)*

1. **What are some things that get in the way of you carrying Narcan?**

[*Can ask hypotheticals if they are mostly in the home with COVID or haven’t obtained Narcan]*

1. **Tell me comfort with giving someone Narcan/naloxone for an overdose?**
2. **Have you ever been in a situation where you or someone you know needed naloxone for an overdose?**

*Yes, no, prefer not to say*

*If comfortable, would you share what the experience was like for you?*

**8. What other information do you want us to know about your experiences with Narcan?**
